# FOXP3, a novel glioblastoma oncosuppressor, affects proliferation and migration

**DOI:** 10.18632/oncotarget.644

**Published:** 2012-09-22

**Authors:** Véronique Frattini, Federica Pisati, Maria Carmela Speranza, Pietro Luigi Poliani, Gianmaria Frigé, Gabriele Cantini, Dimos Kapetis, Manuela Cominelli, Alessandra Rossi, Gaetano Finocchiaro, Serena Pellegatta

**Affiliations:** ^1^ Unit of Molecular Neuro-Oncology, Fondazione I.R.C.C.S. Istituto Neurologico C. Besta, Milan, Italy; ^2^ Service of Bioinformatics, Fondazione I.R.C.C.S. Istituto Neurologico C. Besta, Milan, Italy; ^3^ Department of Experimental Oncology, European Institute of Oncology - Campus IFOM-IEO, Milan, Italy; ^4^ Department of Pathology, University of Brescia, Brescia, Italy

**Keywords:** FOXP3, glioblastoma, neurospheres, proliferation, migration

## Abstract

The transcription factor FOXP3 plays an essential role in regulatory T cell development and function. In addition, it has recently been identified as a tumor suppressor in different cancers. Here, we report that FOXP3 is expressed in normal brain but strongly down-regulated in glioblastoma (GB) and in corresponding GB stem-like cells growing in culture as neurospheres (GB-NS), as evaluated by real time-PCR and confirmed by immunohistochemistry on an independent set of GB. FOXP3 expression was higher in low-grade gliomas than in GB. Interestingly, we also found that neurosphere generation, a feature present in 58% of the GB that we examined, correlated with lower expression of FOXP3 and shorter patient survival. FOXP3 silencing in one GB-NS expressing measurable levels of the gene caused a significant increase in proliferation and migration as well as highly aggressive growth in xenografts. Conversely, FOXP3 over-expression impaired GB-NS migration and proliferation in vitro.

We also demonstrated using ChiP that FOXP3 is a transcriptional regulator of p21 and c-MYC supporting the idea that dysregulated expression of these factors is a major mechanism of tumorigenesis driven by the loss of FOXP3 expression in gliomas. These findings support the assertion that FOXP3 exhibits tumor suppressor activity in glioblastomas.

## INTRODUCTION

The forkhead transcription factor FOXP3 plays an essential role in the development and function of regulatory T cells (Treg), defined as FOXP3^+^CD4^+^CD25^+^ T cells [1]. In humans, FOXP3 is present in two isoforms, referred to as a and b, but the functional differences between the two isoforms are still unclear [2, 3]. A recent report found that in melanomas, FOXP3 is also expressed by tumor-reactive CD8^+^ T cells. These lymphocytes do not express regulatory markers and maintain early effector profiles (CD38^+^, T-bet^+^, perforin^+^) [4]. The expression of FOXP3, however, is not restricted to lymphoid tissues such as the thymus, spleen and lymph nodes. It was recently reported that FOXP3 is expressed in tumor cells from pancreatic carcinoma, breast cancer, melanoma, lung cancer and colon cancer [5]

FOXP3 appears to be a multifaceted factor with seemingly opposite functions in cancer biology. In pancreatic carcinoma and in melanoma, FOXP3 has a tumor-enhancing role through Treg and their effect on tumor tolerance [6, 7]; in ovarian, breast and prostate cancer, FOXP3 has a tumor-suppressing function [8, 9]. In breast and prostate cancer, FOXP3 may modulate the expression of oncogenes or tumor suppressor genes, including ERBB2, SKP2, c-Myc, p21 and other important cancer-related genes [9-11]. Finally, adult T-cell leukemia/lymphoma cells from blood and skin tumors express FOXP3 at high levels but lack suppressor activity, suggesting that in these cells, despite their derivation from the immune system, the role of FOXP3 is unrelated to immune escape [12].

We investigated FOXP3 expression in normal brains and in gliomas, the most frequent of primary brain tumors. We focused our studies on glioblastoma (GB), the most malignant glioma, and GB stem-like cells, the GB subpopulation relevant for tumor perpetuation [13]. A growing number of data has been obtained in recent years, contributing to an improved definition of the GB genome [14]. The identification of mutations of isocytrate dehydrogenase 1 (IDH1) has been particularly relevant [15], contributing to novel efforts aimed at therapeutic targeting of the GB genome [16]. Another example is provided by the identification of increased copy number of TACC3, an Aurora-A kinase substrate [17]: we have recently collaborated to the identification of a fusion protein of TACC3 with fibroblast growth factor receptor with constitutive kinase activity, triggering aneuploidy in GB cells [18]. Here we have found that FOXP3 is involved in modulating the biological properties of GB stem-like cells, such as proliferation and migration, by activation of p21 and repression of c-MYC expression.

## RESULTS

### FOXP3 is strongly down-regulated or absent in glioblastoma

In the initial experiments shown in Figure S1, we found Foxp3+ cells in murine malignant gliomas derived from the GL261 cells. Foxp3 expression was detectable in the early stages of tumor development (10 days after the intracranial injection) but disappeared with tumor progression (20 days after the intracranial injection) (Figure S1A-B). An immunofluorescence analysis confirmed that the Foxp3+ cells were not of immune origin (Figure S1C).

**Figure 1:**
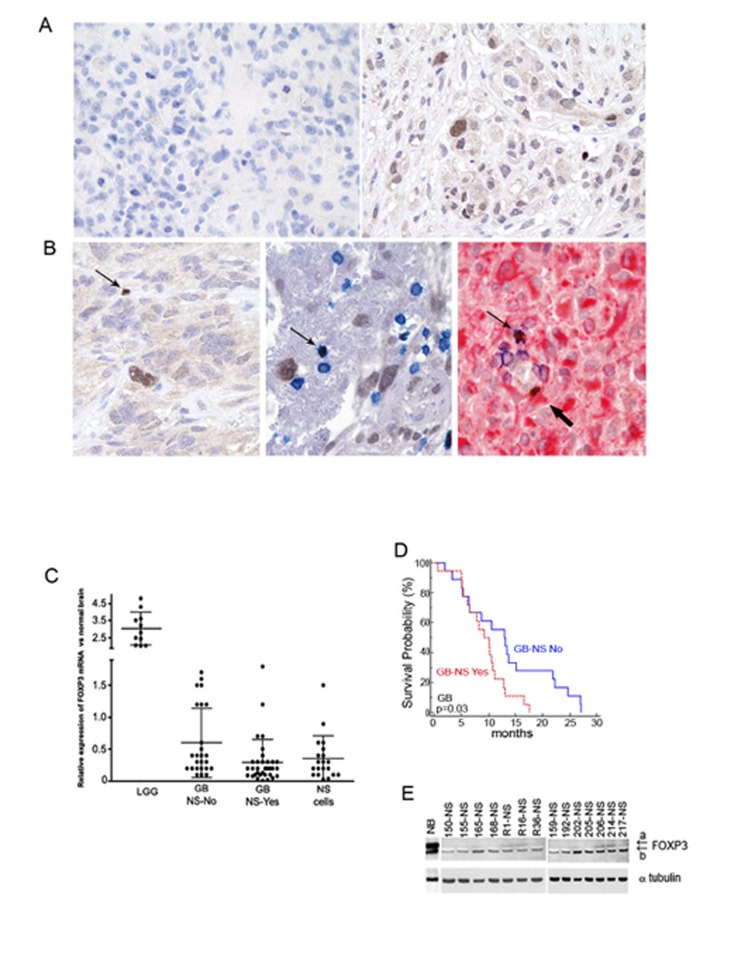
FOXP3 is strongly down-regulated in GB specimens and in corresponding GB-NS lines A) Two representative GB specimens labelled with anti-human FOXP3 antibody (magnification 20X). The section on the left shows one GB completely negative for FOXP3 reactivity. The section on the right shows one GB with FOXP3+ cells mainly concentrated around blood vessels. B) Left panel. FOXP3 staining is detected in one cell with lymphocyte morphology (small and round nucleus; arrow, brown) and in one cell with the morphologic features of tumor cells (large, irregular nucleus, brown). Central panel: Infiltrating lymphocytes identified using CD3 as the marker are localized near blood vessels (thin arrow, blue). Right panel: one FOXP3+ GFAP+ tumor cell (thick arrow, red) is located around a blood vessel together with a CD3+ lymphocyte (thin arrow) (magnification 40X). C) Real-time PCR analysis was performed on 11 LGG, 59 GB and 20 GB-NS. Normal brain was defined as 1.0. The GB specimens were divided into GB-NS-Yes (34/59) and GB-NS-No (25/59) based on their capacity to give rise to NS. GB-NS-Yes expressed less FOXP3 compared to GB-NS-No (P = 0.03). GB-NS shows FOXP3 down-regulation, with the exception of one line (BT165-NS). D) NS formation is associated with shorter survival. A Kaplan Meier survival analysis showed that the median survival of patients with GB forming NS (GB-NS-Yes; n = 18) was lower than that of other patients (GB-NS-No; n = 18) (9.2 months vs. 13.0 months; P = 0.03). E) A western blot shows FOXP3 in GB-NS lines derived from primary (11 lines) or relapsing GB (3 lines) and in normal brain (NB). FOXP3 isoform a is weakly detected in all cases.

We then investigated the expression of FOXP3 in human GB specimens. An immunohistochemistry analysis of 35 GB showed variable expression of FOXP3 (Table S1), with most of the specimens displaying complete absence or scarcity (less than 20%) of FOXP3+ cells: only 4 of 35 GB (11%) showed moderate or strong staining for FOXP3 (Table S1 and Figure 1A and B). By histological analysis, we detected positive nuclear staining for FOXP3 not only in small lymphocytes but also in cells with neoplastic features, such as irregular hyperchromatic nuclei (Figure 1B left panel). To ascertain the identity of FOXP3+ cells, we performed combined immunostaining for FOXP3, CD3 and GFAP. FOXP3+ tumor cells were identified by GFAP expression and negativity for the CD3 marker (Figure 1B central and right panel).

We used real-time PCR to evaluate the mRNA expression of FOXP3 in 11 low-grade gliomas (LGG), 59 GB (55 primary and 4 recurrent) and 20 GB primary cell lines growing in culture as neurospheres (NS) (Figure 1C). FOXP3 expression was significantly higher in LGG in GB (mean ± SD: 0.4 ± 0.4 fold, P < 0.0001 vs. normal brain). GB may or may not give rise to NS (GB-NS-Yes and GB-NS-No, respectively): 58% of these tumors (34/59) were GB-NS-Yes and had lower FOXP3 expression compared to GB-NS-No (mean ± SD: 0.3 ± 0.3 vs. 0.6 ± 0.5, respectively, P = 0.03). The overall survival (OS) of patients with GB-NS-Yes and lower FOXP3 expression was significantly shorter than the OS of patients with GB-NS-No and higher FOXP3 expression (P = 0.03 by Kaplan Meier analysis; Figure 1D). These patients (n= 36) had all been treated by surgery, radiotherapy and chemotherapy with temozolomide [19]. In addition, we analysed FOXP3 expression by real-time PCR in 20 GB-NS. The mean expression compared to normal brain was 0.4 ± 0.3 (P < 0.0001 vs. normal brain). Only BT165-NS and the corresponding specimen expressed FOXP3 at higher levels than normal brain (1.8 and 1.5 fold change vs. control, respectively; top dot in the plot showing NS, Figure 1C).

These data were in agreement with a western blot analysis performed on 14 NS primary cell lines, 11 cell lines derived from primary GB and 3 cell lines from recurrent GB with normal brain lysate as a control (Figure 1E). Six NS cell lines expressed higher levels of FOXP3 than the others. Of these cell lines, BT165-NS grew faster than the other five cell lines and was used for further experiments. Isoform a was weakly present or absent, as also found in immortalized and malignant mammary epithelial cell lines that preferentially express the FOXP3 isoform b (Figure S2) [20].

### FOXP3 is differentially expressed in normal brain

**Figure 2:**
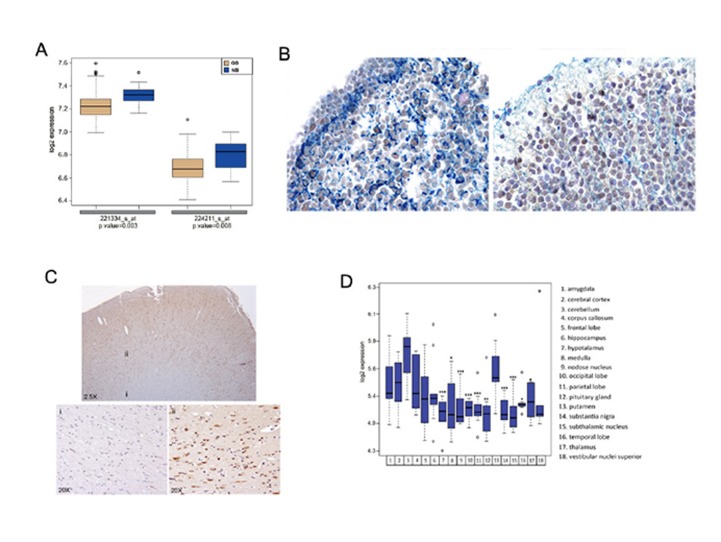
FOXP3 is expressed in human fetal and adult normal brain A) Boxplots represent FOXP3 expression reported on the y-axis as the log2-transformed probe set intensity in GB compared to NB (GSE4290 dataset). The probe set intensity signal represents the amount of FOXP3 mRNA. The boxes are drawn from the 25^th^ and 75^th^ percentiles in the distribution of FOXP3 intensity. The median FOXP3 expression is higher in NB than in GB (see also Methods in Supplementary Data). B) FOXP3 expression in a normal fetal brain. Combined FOXP3 and GFAP double staining was carried out on the periventricular zone (left) and on the cortical layer (right). C) FOXP3 immunohistochemistry was performed on adult human brain obtained by Biochain (Hayward, CA USA). The upper panel shows the brain area investigated (2.5X); the lower panels show the white matter (i) and the cortical area (ii) (20X). D) Boxplots represent the log2-transformed, probe set intensity of FOXP3 (221333_at probe set) categorized for 18 brain areas. The probe set intensity signal is the measure of FOXP3 mRNA. The probe set 221333_at that targets FOXP3 was significantly up-regulated in the putamen compared to the other 10 areas (* P < 0.05; ** P < 0.01; *** P < 0.005). All P values were calculated using a t-test, except for the P values of the occipital lobe and the temporal lobe, which were obtained using a Wilcoxon test (Method in Supplementary Data).

FOXP3 expression has not been observed in normal brain to date. We first used the public microarray dataset GSE4290 (http://www.ncbi.nlm.nih.gov/geo/query/acc.cgi?acc=GSE4290) to compare FOXP3 expression in human normal brain and in GB. After a quality control evaluation, we selected 88/105 samples; 17 outliers were excluded. An analysis with the microarray GSE4290 dataset of two probe sets corresponding to the FOXP3 gene (221334_s _at and 224211_s_at) confirmed that the expression of FOXP3 is significantly down-regulated in 73 glioblastomas compared to 15 normal brains (P = 0.003 for 221334_s _at; P = 0.008 for 224211_s_at; Figure 2A). We then investigated FOXP3 expression by immunohistochemistry in five-month-old human fetal brain. FOXP3+ cells were found in the periventricular zone (Figure 2B, left) and the cortical area (Figure 2B, right). Of note, in the periventricular zone, many cells co-expressed FOXP3 and GFAP, while in the cortical layer, most cells were only positive for FOXP3. In the adult brain, several FOXP3+ cells were found in cortical areas (Figure 2C, lower right), while the white matter was negative (Figure 2C, lower left).

We also analysed FOXP3 expression in the public GSE3526 microarray dataset (http://www.ncbi.nlm.nih.gov/geo/query/acc.cgi?acc=GSE3526). After quality controls, 138/151 samples were selected. FOXP3 was differentially expressed in the 18 brains represented in the data set and significantly up-regulated in the cerebellum and the putamen (Figure 2D).

### FOXP3 affects glioblastoma proliferation and migration both in vitro and in vivo

**Figure 3:**
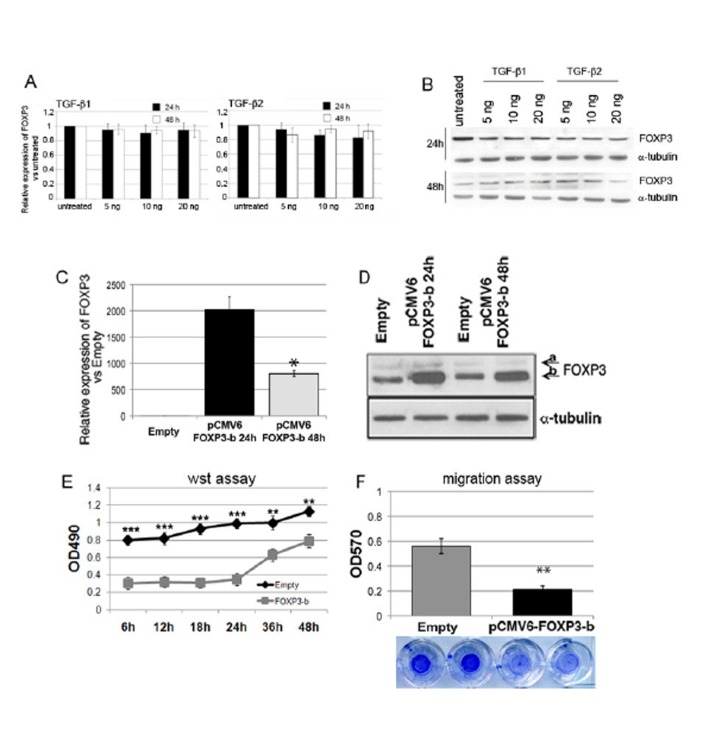
Modulation of FOXP3 expression affects the proliferation and migration of GB-NS A and B) Three GB-NS lines (BT150-NS, BT165-NS, BT168-NS) were treated with 5, 10, or 20 ng/ml of TGF-β1 or TGF-β2 for 24 h or 48 h. The FOXP3 transcript was evaluated by real-time PCR. Histograms represent the mean ± SD of three different GB-NS lines. C) The relative expression of FOXP3-b vs. empty after transfection with pCMV6 retroviral vector shows a significant increase in FOXP3-b levels (2022.6 ± 240.1 fold vs. empty vector at 24 h), decreasing significantly at 48 h (804.2 ± 56.9 48 h fold vs. empty vector (*P* < 0.0005 vs. 24 h). D) A western blot displays a significant increase of FOXP3 in FOXP3-b pCMV6 cells compared to empty cells. Alpha-tubulin was used as the control. The data for FOXP3 over-expression are based on a representative cell line; this experiment was performed using four GB-NS. E) The proliferation analysis indicates that pCMV6 FOXP3-b cells proliferate significantly less than empty cells (* P < 0.01). F) The migration assay shows a significant decrease in pCMV6 FOXP3-b cells vs. empty cells (** P < 0.001).

To study the regulation of FOXP3 expression in GB, we first tested the effects of TGF-β, because this factor is a major regulator of FOXP3 expression in T lymphocytes [21, 22]. TGF-β1 and TGF-β2 did not influence FOXP3 mRNA and protein levels in three GB-NS (Figure 3A and B). We then investigated the biological effects of the modulation of FOXP3 expression by either over-expression by vector transfection or silencing via a lentiviral vector.

To investigate their specific role, FOXP3 isoforms a and b were separately over-expressed in GB-NS. The over-expression was confirmed by real-time PCR and western blot analysis using the empty vector as the internal control (Figure 3C and D). FOXP3-b was significantly reduced 48 h after transfection (P < 0.001, Figure 3C); a similar significant reduction was observed for FOXP3-a (Figure S3A). We then verified the impact of FOXP3 up-regulation on proliferation and migration by performing in vitro proliferation and migration assays. Reduced proliferation was observed when the FOXP3 isoform b (Figure 3E) or isoform a (Figure S3B) was over-expressed (P < 10^7^ from 6 to 24 h; P = 0.001 at 36 and 48 h). Differences in the proliferation rates decreased after 24 h in parallel with FOXP3 expression. Over-expression of FOXP3 b but not a was associated with a significant reduction of migration compared to the empty vector (P = 0.003) (Figure 3F and Figure S3B, respectively). The GB-NS in which isoforms a and b were overexpressed displayed phenotypic changes. In particular, we observed rare neurospheres and single cells attached to the plate (data not shown).

**Figure 4:**
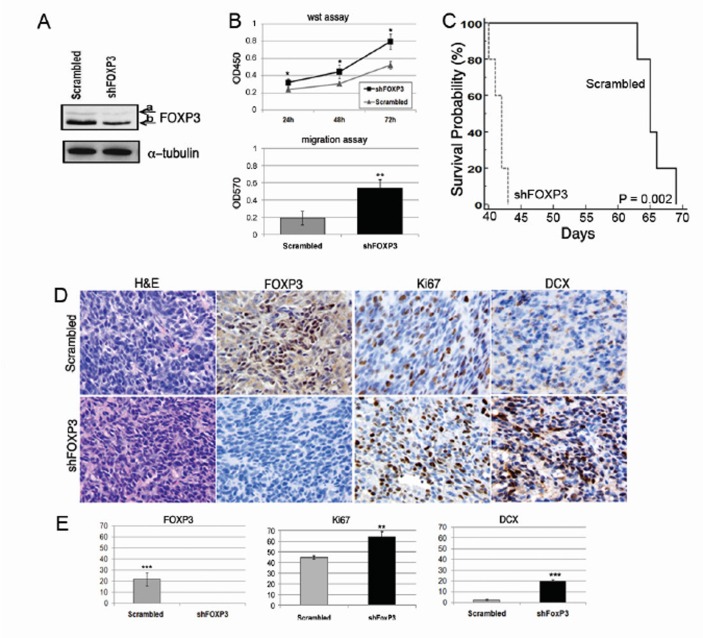
FOXP3 silencing affects migration and proliferation in vitro and in vivo A) Western blot analysis of FOXP3 expression in BT165-NS after infection with the shFOXP3 lentiviral vector reveals a 59% reduction of FOXP3 compared to scrambled; i.e., containing casual non-specific shRNA. FOXP3 expression was normalized with alpha-tubulin and measured with the ImageJ setting the scrambled FOXP3 level at 100%. Alpha-tubulin was used as the control. B) Upper panel: proliferation analysis after FOXP3 silencing. shFOXP3-NS proliferate significantly more than scrambled-NS (* P < 0.05). Lower panel: migration of shFOXP3-NS vs. scrambled NS. Significantly increased migration was observed in shFOXP3 cells vs. scrambled cells (** P < 0.01). C) Kaplan Meier survival analysis of immunodeficient mice injected with 105 BT165-NS transduced with shFoxp3 lentiviral vector or scrambled lentiviral vector (n = 5 per group). D) Immunohistochemistry (evaluations on five 40X independent fields) showed that gliomas from scrambled-NS contain clusters of tumour cells with nuclear FOXP3 staining, while shFOXP3 gliomas were negative. The shFOXP3 tumors have a higher proliferation index (Ki67) and a higher positivity for DCX compared to scrambled tumors. E) Histograms represent the quantification of immunohistochemical staining for FOXP3 (0.0 ± 0.0 % cells in shFOXP3 tumour vs. 21.8 ± 5.9 % in scrambled tumors), Ki67, and DCX positive cells in shFOXP3 and scrambled tumors (* P < 0.01; *** P < 0.0001, **** P < 0.000001). Three mice for each group were studied, and representative images for each tumor are displayed.

FOXP3 silencing was studied in BT165-NS cell line, which expresses measurable levels of FOXP3 and has an adequate proliferation rate that allows in vitro propagation. We obtained a 59% decrease of FOXP3 protein as shown by immunoblotting (Figure 4A); decreased FOXP3 expression was also confirmed by real time-PCR (not shown). The effects of FOXP3 inhibition on proliferation and migration were then evaluated in shFOXP3-NS and in scrambled-NS as the control (Figure 4B, upper panel). Proliferation assays performed at 24 h, 48 h and 72 h showed that shFOXP3-NS proliferate significantly more than scrambled-NS (24 h P <0.01; 48 h P =0.02, 72 h P = 0.04). shFOXP3-NS also had a significantly higher migration capacity compared to scrambled cells (2.8 fold vs. scrambled cells, P = 0.003; Figure 4B, lower panel). In vivo, we found that mice injected with shFOXP3-NS survived significantly less than mice injected with scrambled-NS (mean ± SD: 41.6 ± 1.1 vs. 65.6 ± 2.2 days, P = 0.002; Figure 4C). Histology and immunohistochemistry of the tumors revealed an absence of FOXP3+ cells in shFOXP3 tumors (P < 0.0001), higher proliferation as measured by Ki67+ cells (P < 0.01), and higher migration ability, as evaluated by the identification of cells positive for doublecortin (DCX) in tumors originated by shFOXP3-NS compared to scrambled tumors (P < 0.001; Figure 4D and E). Overall, these data demonstrate that FOXP3 negatively affects proliferation and migration.

### FOXP3 is a transcriptional regulator of p21 and c-MYC in GB-NS

FOXP3 was shown to be involved in the induction of several tumor suppressors, including p21, p18, LAT2, and ARHGAPS in breast cancer [11]. In addition, FOXP3 was reported as to be a repressor of the oncogene c-MYC in prostate cancer [9]. We have focused our validation on p21 as a negative regulator of cell growth [23] and on c-MYC, involved in regulating proliferation and survival of glioma cancer stem cells [24, 25].

**Figure 5:**
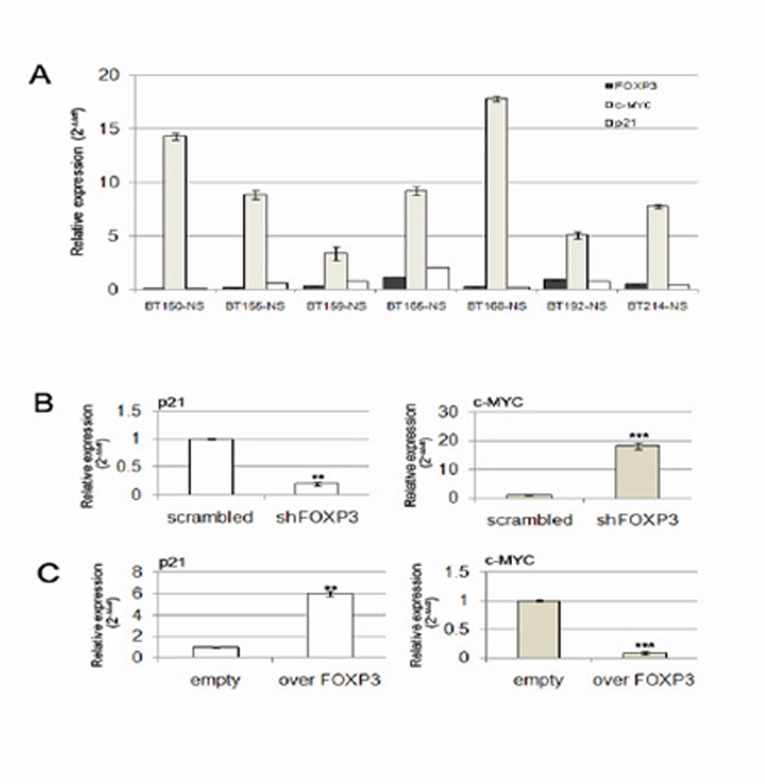
FOXP3 is a transcriptional regulator of p21 and c-MYC A) RT-PCR showed that c-MYC expression is high in GB-NS expressing low levels of FOXP3 and p21. B) A decrease in p21 and an increase in c-MYC expression is observed when FOXP3 is silenced. C) Induction of p21 and reduction of c-MYC expression are induced by FOXP3 over-expression. (** P < 0.005, *** P < 0.001)

In order to directly demonstrate the FOXP3-mediated induction of p21 and repression of c-MYC, we performed a ChIP assay on GB-NS. We found that FOXP3 specifically binds the p21 transcription start site (TSS). Interestingly, specific binding of FOXP3 was also demonstrated for the TSS of c-MYC (Figure S4). We then evaluated the expression levels of FOXP3, p21 and c-MYC in 7 GB-NS lines and found that the expression of FOXP3 and p21 was weak or absent in the presence of c-MYC up-regulation (Figure 5A). To investigate further the relationship of p21, c-MYC and FOXP3 expression, we analysed p21 and c-MYC levels in BT165-NS after FOXP3 silencing or over-expression. We found a significant reduction of p21 and an increase of c-MYC expression in shFOXP3 cells (P < 0.001 and P < 0.0001 compared to scrambled cells, respectively; Figure 5B). On the contrary, over-expression of FOXP3 isoform b caused a significant increase of p21 and a strong down-regulation of c-MYC expression compared to empty cells (P < 0.0001; Figure 5C). We did not find differences in p21 and c-MYC levels by over-expressing isoform a (data not shown).

These data support the evidence that FOXP3 is a direct transcriptional regulator for p21 and c-MYC.

## DISCUSSION

Recent reports demonstrate that FOXP3 is expressed in non-lymphocytic cells, suggesting that its expression and function are not restricted to the T-cell lineage. Normal pancreatic ducts cells are devoid of FOXP3 expression, which has been detected in human pancreatic cancer cells [6]. FOXP3 is also expressed in human melanoma cells but not in normal melanocytes [7]. In contrast, in the breast and prostate, FOXP3 is expressed in normal epithelial cells but down-regulated in corresponding cancer cells [9, 10]. In the present study, we provide evidence of down-regulation of FOXP3 expression in both primary and recurrent GB specimens and in corresponding cell lines growing as neurospheres.

We found that GB-NS express both isoforms, but isoform b is more expressed than isoform a. Treg cells co-express equal amounts of the two isoforms. Moreover, equal amounts of isoform a are localized in the cytoplasm and the nucleus, while isoform b, lacking the nuclear export signal, is primarily located within the nucleus. After specific stimulation, isoform a preferentially locates into the cytoplasm of activated T-cells [26], where it can bind NFkB and prevent its localization to the nucleus following activation stimuli [27]. This is relevant, given the important role that the NFkB signalling pathway and its target genes play in GB progression [28].

The overexpression of isoform a and b separately resulted in a similar strong suppression of proliferation, with a less potent reduction of migration in the presence of isoform a.

Notably, we show for the first time that FOXP3 is expressed in normal brain, supporting further research on the potential role of FOXP3 in brain development.

The major finding of this study, however, is the contribution of FOXP3 down-regulation to GB biology. We first verified that FOXP3 expression in GB-NS is not modulated by TGF-β, a factor playing a central role in the maintenance of FOXP3 expression in Treg cells [21, 29], suggesting that FOXP3 expression in GB-NS is not related to immune escape. Instead, our data, obtained by modulating FOXP3 expression, establish a role for FOXP3 in modulating the biological properties of GB stem-like cells, including proliferation, migration and in vivo aggressiveness.

Downstream FOXP3, we found that down-regulation of p21, a protein involved in stem cell differentiation and apoptosis [23], is associated with FOXP3 down-regulation, as recently reported in breast cancer samples [11]. We also confirmed that FOXP3 is a direct transcriptional regulator of c-MYC, as described in prostate cancer [9]. c-MYC plays a role in the survival and maintenance of GB stem-like cells and is considered a central gene implicated in genetic reprogramming [30, 31]. Moreover, c-MYC is one of seven genes whose expression is associated with worse prognosis in GB [32]. In one of the mouse models developed to study glioma origin [33], c-myc expression under the GFAP promoter in developing astroglia predisposes to malignant gliomas [34]. These tumors seem to originate from GFAP expressing cells in the ventricular zone, indicating that during astrocytic development, c-myc overexpression is sufficient to promote a neoplastic process by inducing the proliferation of early astroglial cells. This scenario and our results, in particular the presence of periventricular FOXP3/GFAP+ cells in normal fetal brains, support the hypothesis that during astrocytic development, and possibly during astrocytic proliferation in reactive gliosis [35], FOXP3 orchestrates the induction of astroglial terminal differentiation by preventing c-myc activation and proliferation in glial cell precursors.

Further studies are required to investigate the molecular mechanisms responsible for FOXP3 down-regulation in glioblastomas. Phosphatidylinositol 3 kinase (PI3K) signalling, necessary to stimulate glioma invasion and migration [36, 37] downregulates FOXP3 expression by sequestering FOXO1 and FOXO3a factors in the cytoplasm [38]. Thus, a thorough investigation of the role of this pathway in down regulation of FOXP3 expression in GB is of interest, also considering that FOXO3a is an important regulator of differentiantion and tumorigenicity of GB-NS [39]. Dysregulation of the epigenetic control of FOXP3 expression can also play a role in downregulation of FOXP3 expression [40]. Polycomb histone methyltransferease can silence the FOXP3 promoter [41] through the action of its catalytic subunit EZH2, that we and others have found upregulated in GB and malignant gliomas [42], [43]. Furthermore, DNA methyltransferase DNMT1, involved in the maintenance and self-renewal of progenitor cells in somatic tissues [44], and DNMT3B are up-regulated in gliomas [45]. The inhibition of DNMT1 and DNMT3B in T-cells leads to FOXP3 expression, suggesting another pathway of FOXP3 regulation by epigenetic modification.

## MATERIAL AND METHODS

### Tumor specimens and cell cultures

Primary glioblastomas (GB), recurrent glioblastomas (GBMR) and grade II gliomas, including oligoastrocytomas, fibrillary and gemistocytic astrocytomas (low-grade gliomas, LGG), were obtained from the department of Neurosurgery of the “Istituto Neurologico Carlo Besta” after the informed consent of the patients was obtained. Glioma specimens were frozen and/or placed in a saline solution after surgery. GB cell lines were obtained after dissociation in collagenase type I (Invitrogen-Life Technologies, Carlsbad, California, USA) and grown as neurospheres (GB-NS) in DMEM/F12 (GIBCO- Life Technologies, Carlsbad, California, USA) containing penicillin-streptomycin (1:100, EuroClone, Milan, Italy), B-27 (1:50, GIBCO- Life Technologies), human recombinant fibroblast growth factor 2 (bFGF; 20 ng/mL; Tebu-bio, Milan, Italy), and epidermal growth factor (EGF; 20 ng/mL; Tebu-bio).

### RNA extraction and Real-Time PCR

Total RNA was extracted using Trizol (Life Technologies, Rockville, Maryland, USA) from human snap frozen tissues and human GB-NS. Total RNA was reverse-transcribed using a High Capacity cDNA Synthesis KIT (Applied Biosystems-Life Technologies, Carlsbad, California, USA). The expression of FOXP3 was analysed by real-time PCR TaqMan chemistry, performed on an ABI PRISM 7900 real-time PCR system (Applied Biosystems, Foster City, CA, USA). The FOXP3 primer (FAM dye-labelled) was provided by an on-demand TaqMan Gene expression Assay (Hs00203958_m1, Applied Biosystems). Beta-2-microglobulin (Hs99999907_m1, Applied Biosystems) was chosen as the reference gene, and commercial RNA from a normal human brain (Life Technologies) was used as the calibrator for the calculation of fold expression levels with the ΔΔCt method. The RNA inputs were normalized against beta-2-microglobulin.

### Western blot analysis and antibodies

Membranes with transferred proteins were incubated with a primary antibody; either anti-FOXP3 antibody (1:250; eBioscience, Science Center Drive, San Diego, USA) or anti-alpha-tubulin antibody (1:5000). The primary antibody interaction was followed by incubation with peroxidase conjugated to the secondary antibody [anti-rat (1:10000), anti-mouse (1:10000) or anti-rabbit (1:10000)]. Chemoluminescence was detected using the ECL (enhanced chemiluminescence) Plus kit (Amersham, GE Healthcare). Human normal brain tissue lysate (GeneTex, Inc., Irvine CA) was used as the control.

### Silencing and over-expression of FOXP3

The cells were transduced with lentiviral particles (MISSION shRNA Lentiviral Vectors, Sigma Aldrich, St. Louis, Missouri, USA) containing FOXP3 specific shRNA sequences (shFOXP3) according to the manufacturer's recommendations. Five different FOXP3-specific sequences were screened, and the most efficient sequence was chosen. As a negative control, we used shRNA Lentiviral Particles encoding non-specific shRNA (scrambled cells). Four days after infection, cells were selected for puromycin resistance (1.2 μg/ml) for one week.

Two pCMV6-FOXP3 vectors (OriGene Technologies, Inc., Rockville, MD) were used to transfect GB stem-like cells for FOXP3 over-expression: variant 1 (isoform a) or variant 2 (isoform b). The pCMV6-empty vector was used as the negative control.

### In vivo experiments

Sixteen immune-deficient CD1-nude mice received a brain injection of 10^5^ “silenced” or “scrambled” cells (n = 5/group for survival, n = 3/group for histological studies). The stereotactic coordinates with respect to the bregma were 0.7 mm posterior, 3 mm left lateral, and 3.5 mm deep into the nucleus caudatum. The animals were monitored every day until they were euthanized, in accordance with the current directives of the Campus animal IFOM-IEO house facility, the Ethics Committee of the Institution and the Minister of Health.

### Proliferation and migration Assay

The Cell Proliferation Reagent WST-1 (Roche Applied Science, Hague Road Indianapolis USA) was used to test for GB-NS proliferation and was performed by plating 5000 cells/well, as suggested by the manufacturer. Eight replicates per point were completed. In vitro migration was assayed using the Transwell-96 system (BD Bioscience, Qume Drive San Jose, CA, USA) as provided by the manufacturer. After 24 h, migrated cells were stained with crystal violet solubilised with 10% acetic acid.

### Immunohistochemistry and immunofluorescence

Paraffin was removed with xylene and the sections were rehydrated in graded alcohol. Antigen retrieval was carried out using preheated target retrieval solution (pH 6.0), and the primary antibodies were incubated overnight. The following antibodies were used: FOXP3 (eBiosciences; 1:40), Ki67 (BD Bioscience; 1:50), and CD3 (1:100; Thermo Scientific, Wyman Street Waltham MA, USA). Single immunostains were performed using a standard immunoperoxidase protocol (Vectastain Elite ABC kit, PK-6100; Vector Laboratories, Inc., Burlingame, CA, USA) followed by a diaminobenzidine chromogen reaction (Peroxidase substrate kit, DAB, SK-4100; Vector Lab). The tumor sections were also stained with hematoxylin and eosin to assess the volume of tumor growth. Bright field combined immunostains were performed using the rat-on-mouse HRP-Polymer Kit (Biocare Medical, Pike Lane Concord, CA, USA) for the detection of FOXP3 or the MACH4 Universal AP Polymer Kit (Bio care Medical) for the detection of CD3 and GFAP. The chromogen reaction was developed by DAB, Ferranti Blue or Alkaline Phosphatase/RED, Rabbit/Mouse (DAKO), and the nuclei were counterstained with methyl green. For the double immunofluorescence analysis, tumor sections were incubated with Alexa Fluo-conjugated antibodies for 1 h, counterstained with DAPI (4′,6-diamidino-2-phenylindole, Sigma), and examined using a LEICA SP2 confocal microscope.

### Statistical analysis

Cumulative survival curves were constructed by Kaplan–Meier method (MedCalc 9.3). Statistical comparisons of data sets were performed by Student's two-tailed t-test, and the results were considered significant at P < 0.05.

## Supplementary Figures and Tables


