# Supplementary material for: FOXP3, a novel glioblastoma oncosuppressor, affects proliferation and migration
Source: Oncotarget. 2012 Sep 22;3(10):1146–57. doi: 10.18632/oncotarget.644 (PMC3717952; doi:10.18632/oncotarget.644)
Supplement: Supplementary file 1 [file oncotarget-03-1146-s001.pdf]

FOXP3, a novel glioblastoma oncosuppressor, affects proliferation and migration-Frattini et al

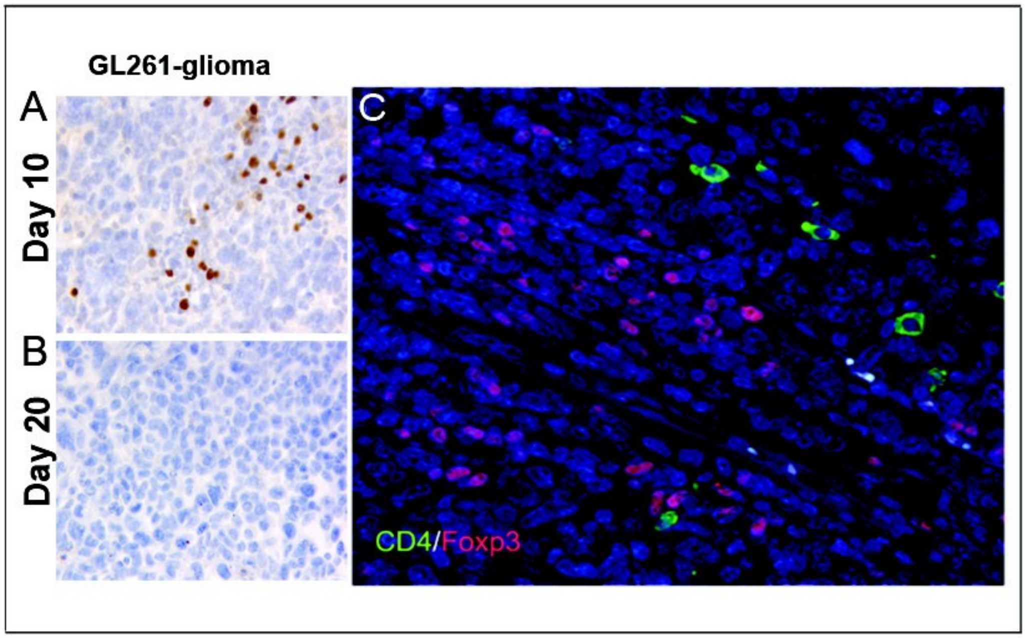

Figure S1. Foxp3 expression in murine GL261 glioma during tumor development.
A) GL261 glioma 10 days after tumor implantation: Foxp3 positive cells present morphological features of tumor cells with large and irregular nuclei (magnification 40X). B) GL261 glioma 20 days after tumor implantation: Foxp3 positive cells are absent (magnification 40X).
C) Immunofluorescence of Foxp3 and CD4 staining of paraffin-embedded GL261 glioma: Foxp3$^+$ cells (red) and CD4$^+$ cells (green). We detected Foxp3 positive cells into the tumor mass while CD4 positive cells are mainly disseminated along a blood-vessel. DAPI staining (blue) highlights the abnormal number of nuclei per cell, a characteristic of tumor cells.

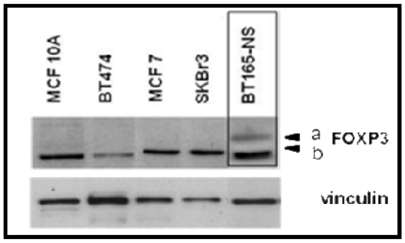

Supplementary Figure S2: Characterization of FOXP3 in primary NS cell lines compared to Immortalized and Malignant Mammary Epithelia Cells.

Western Blot shows FOXP3 protein level in BT165-NS compared to immortalized (MCF10A) and malignant (BT474, MCF7 and SKBr3) mammary epithelial cells. Vinculin has been used as a loading control. These cell lines preferentially express the FOXP3 isoform b, as reported by Zuo et al [1]. BT165–NS cell line reported in Figure S2 expresses moderate levels of isoform a.

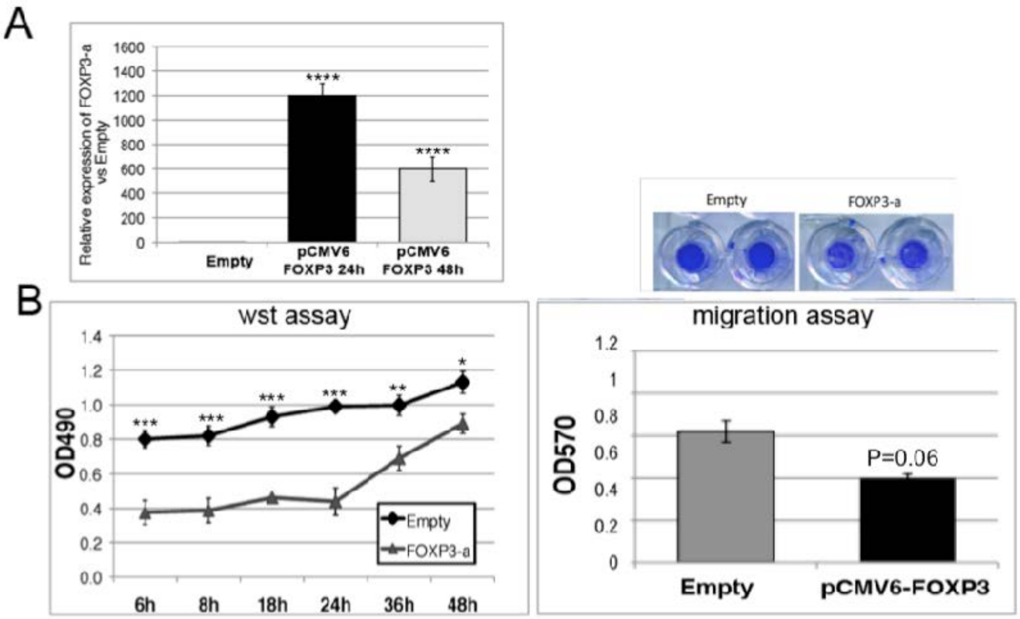

Figure S3: Effects of FOXP3 a overexpression on proliferation and migration.

A) Relative expression of FOXP3 after transfection of the FOXP3-a construct increased at 24h after transfection ($P = 10^{-6}$). B) Left panel. Proliferation of BT165-NS cells transfected with pCMV6 FOXP3-a is slower than that of empty cells (*** $P < 0.0001$, ** $P < 0.005$, * $P < 0.01$). Right panel. Migration assay shows that the decrease in cell migration of pCMV6 FOXP3-a cells vs empty cells is not significant ($P = 0.06$).

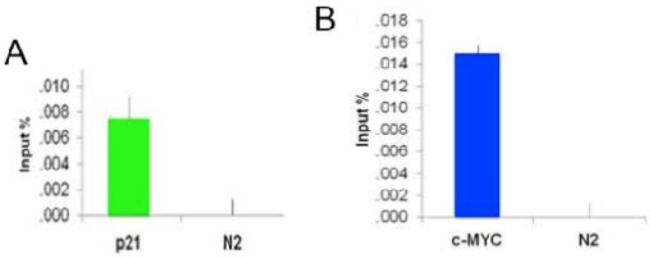

Figure S4: Binding of FOXP3 to p21and c-myc Transcription Starting Sites (TSS) in GB-NS. A-B) Nuclear preparations from BT165-NS were fixed with paraformaldehyde. After sonication, genomic DNA associated with FOXP3 was immune-precipitated and quantified by RT-PCR. The quantity of precipitated DNA was compared with the total input of genomic DNA, amplified by p21 and c-Myc specific primers. Primers mapping on telomeric regions were used as negative control (N2 primers). Non specific binding from control IgG ChIPs was subtracted and data were represented as percent input. Standard deviations from two experimental replicates are shown. NS were processed for qChIP as previously described [2], with few modifications. Briefly, formaldehyde (37%) was added to the culture medium to a final concentration of 1%. Cross-linking was stopped by addition of glycine (0.125 M final concentration). Fixed cells were washed twice with PBS and harvested in SDS buffer (50 mM Tris pH 8.1, 0.5% SDS, 100 mM NaCl, 5 mM EDTA, and protease inhibitors). Cells were pelleted by centrifugation and resuspended in IP buffer (100 mM Tris pH 8.6, 0.3% SDS, 1.7% Triton X-100, and 5 mM EDTA). DNA fragments with a bulk size of 300-500 bp were obtained using a Branson digital sonifier 250 D. For each immune-precipitation, 1 ml of diluted lysate ($5x10^6$ cells/ml) was pre-cleared using protein A beads (50% slurry protein A-Sepharose, Amersham) and immunoprecipitated overnight at 4°C with antibody specific for FOXP3 (ChIP grade antibody from Abcam, UK), in presence of 20ul of magnetic dynabeads. Beads were washed and crosslink was reversed in 1ml of water with 10% Chelex 100 (Bio-Rad, cat. no. 142-1253), and used directly for qPCR. PCR primer sequences:

p21 p21_Fw AGGCACTCAGAGGAGGTGAGA; p21_Rv CAGAAACACCTGTGAACGCA

N2 N2_Fw AGCTATCTGTCGAGCAGCCAAG; N2_Rv CATTCCCCTCTGTTAGTGGAAGG

c-Myc c-Myc_Fw GAAATTGGGAACTCCGTGTG; c-Myc_Rv CTAGGGCGAGAGGGAGGTT

Microarray Dataset analysis.

Microarray data Selection and accession numbers. Two microarray datasets were selected from microarray experiments performed with GeneChips Affymetrix HGU133Aplus 2.0 arrays (Dataset GSE4290; 105 samples – 22 Normal Brain and 82 Glioblastomas) [3] and HGU133Aplus 2.0 arrays (Dataset GSE3526; 151 samples – 18 regions of the central nervous system). All microarray data were available from the ArrayExpress database (http://www.ebi.ac.uk/arrayexpress).

Pre-processing microarray data. All arrays were tested for statistical quality controls provided by the array Quality Metrics Bioconductor package [4]: outlier samples were excluded from the analysis. This package allows users to perform a wide variety of data quality assessment approaches and to identify outlier arrays (i.e., arrays of low quality or very different from the whole dataset), in such a way to enhance statistical and biological significance of the analysis.

The Robust Multichip Average (RMA) [5] algorithm was applied to normalize with quantile method and calculate probeset intensity. Normality of the distribution and homogeneity of variance in our datasets were tested using the Shapiro–Wilk and Bartlett's tests [6]. Differences of FOXP3 expression were studied with the t-test or Wilcoxon together with a false discovery rate correction of the p-value (Bonferroni correction). The corrected p-value threshold cut-off was less or equal to 0.05.

References

1. Zuo T, Wang L, Morrison C, Chang X, Zhang H, Li W, Liu Y, Wang Y, Liu X, Chan MW, Liu JQ, Love R, Liu CG, Godfrey V, Shen R, Huang TH, et al. FOXP3 is an X-linked breast cancer suppressor gene and an important repressor of the HER-2/ErbB2 oncogene. Cell. 2007; 129: 1275-1286.

2. Frank SR, Schroeder M, Fernandez P, Taubert S, Amati B. Binding of c-myc to chromatin mediates mitogen-induced acetylation of histone H4 and gene activation. Genes Dev. 2001; 15: 2069-2082.

3. Sun L, Hui AM, Su Q, Vortmeyer A, Kotliarov Y, Pastorino S Menon J, Walling J, Bailey R, Rosenblum M, Mikkelsen T, Fine HA. Neuronal and glioma-derived stem cell factor induces angiogenesis within the brain. Cancer Cell. 2006; 9: 287-300.

4. Kauffmann A, Gentleman R, Huber W. arrayQualityMetrics--a bioconductor package for quality assessment of microarray data. Bioinformatics. 2009; 25: 415-416.

5. Irizarry RA, Bolstad BM, Collin F, Cope LM, Hobbs B, Speed TP. Summaries of affymetrix GeneChip probe level data. Nucleic Acids Res. 2003; 31: e15.

6. McCall MN, Irizarry RA. Thawing frozen robust multi-array analysis (fRMA). BMC Bioinformatics. 2001; 12: 369.

Table S1. FOXP3 staining in a cohort of 35 GB.

	Number of positive cells per HPF (40x)					Staining intensity of positive cells			
Tumor #	0-5	6-20	21-50	51-70	71-100	0 absent	1+ weak	2+ Moderate	3+ strong
GB1	X					X			
GB2		X					X		
GB3		X					X		
GB4	X					X			
GB5	X					X			
GB6		X					X		
GB7		X						X	
GB8		X					X		
GB9	X						X		
GB10	X						X		
GB11	X					X			
GB12	X					X			
GB13		X					X		
GB14		X						X	
GB15	X					X			
GB16			X				X		
GB17					X				X
GB18				X			X		
GB19		X					X		
GB20	X						X		
GB21	X					X			
GB22	X					X			
GB23	X					X			
GB24	X					X			
GB25	X					X			
GB26		X					X		
GB27	X					X			
GB28		X					X		
GB29		X					X		
GB30	X					X			
GB31	X					X			
GB32	X					X			
GB33				X					X
GB34	X					X			
GB35	X					X			
